# Immunoglobulin A Antibodies Against Myelin Oligodendrocyte Glycoprotein in a Subgroup of Patients With Central Nervous System Demyelination

**DOI:** 10.1001/jamaneurol.2023.2523

**Published:** 2023-08-07

**Authors:** Ana Beatriz Ayroza Galvão Ribeiro Gomes, Laila Kulsvehagen, Patrick Lipps, Alessandro Cagol, Nuria Cerdá-Fuertes, Tradite Neziraj, Julia Flammer, Jasmine Lerner, Anne-Catherine Lecourt, Nina de Oliveira S. Siebenborn, Rosa Cortese, Sabine Schaedelin, Vinicius Andreoli Schoeps, Aline de Moura Brasil Matos, Natalia Trombini Mendes, Clarissa dos Reis Pereira, Mario Luiz Ribeiro Monteiro, Samira Luisa dos Apóstolos-Pereira, Patrick Schindler, Claudia Chien, Carolin Schwake, Ruth Schneider, Thivya Pakeerathan, Orhan Aktas, Urs Fischer, Matthias Mehling, Tobias Derfuss, Ludwig Kappos, Ilya Ayzenberg, Marius Ringelstein, Friedemann Paul, Dagoberto Callegaro, Jens Kuhle, Athina Papadopoulou, Cristina Granziera, Anne-Katrin Pröbstel

**Affiliations:** 1 Department of Neurology, University Hospital Basel and University of Basel, Basel, Switzerland; 2 Departments of Biomedicine and Clinical Research, University Hospital Basel and University of Basel, Basel, Switzerland; 3 Research Center for Clinical Neuroimmunology and Neuroscience Basel (RC2NB), University Hospital Basel and University of Basel, Basel, Switzerland; 4 Departamento de Neurologia, Instituto Central, Hospital das Clínicas HCFMUSP, Faculdade de Medicina, Universidade de Sao Paulo, Sao Paulo, Brazil; 5 Departamento de Oftalmologia e Laboratorio de Oftalmologia (LIM/33), Instituto Central, Hospital das Clínicas HCFMUSP, Faculdade de Medicina, Universidade de Sao Paulo, Sao Paulo, Brazil; 6 Translational Imaging in Neurology (ThINk) Basel, Department of Biomedical Engineering, University Hospital Basel and University of Basel, Basel, Switzerland; 7 Medical Imaging Analysis Center (MIAC), University of Basel, Basel, Switzerland; 8 Department of Medicine, Surgery and Neuroscience, University of Siena, Siena, Italy; 9 Instituto de Medicina Tropical de Sao Paulo, Faculdade de Medicina, Universidade de Sao Paulo, Sao Paulo, Brazil; 10 Charité–Universitätsmedizin Berlin, Corporate Member of Freie Universität Berlin and Humboldt-Universität zu Berlin, Neurocure Cluster of Excellence, Berlin, Germany; 11 Charité–Universitätsmedizin Berlin, Corporate Member of Freie Universität Berlin, Humboldt-Universität zu Berlin, and Max Delbrueck Center for Molecular Medicine, Experimental and Clinical Research Center, Berlin, Germany; 12 Charité–Universitätsmedizin Berlin, Corporate Member of Freie Universität Berlin and Humboldt-Universität zu Berlin, Department of Psychiatry and Neurosciences, Berlin, Germany; 13 Charité–Universitätsmedizin Berlin, Corporate Member of Freie Universität Berlin and Humboldt-Universität zu Berlin, Institut für Integrative Neuroanatomie, Berlin, Germany; 14 Department of Neurology, St Josef-Hospital, Ruhr University Bochum, Bochum, Germany; 15 Department of Neurology, Medical Faculty, Heinrich Heine University Düsseldorf, Düsseldorf, Germany; 16 Center for Neurology and Neuropsychiatry, LVR-Klinikum, Heinrich Heine University Düsseldorf, Düsseldorf, Germany

## Abstract

**Importance:**

Differential diagnosis of patients with seronegative demyelinating central nervous system (CNS) disease is challenging. In this regard, evidence suggests that immunoglobulin (Ig) A plays a role in the pathogenesis of different autoimmune diseases. Yet little is known about the presence and clinical relevance of IgA antibodies against myelin oligodendrocyte glycoprotein (MOG) in CNS demyelination.

**Objective:**

To investigate the frequency of MOG-IgA and associated clinical features in patients with demyelinating CNS disease and healthy controls.

**Design, Setting, and Participants:**

This longitudinal study comprised 1 discovery and 1 confirmation cohort derived from 5 centers. Participants included patients with suspected or confirmed demyelinating diseases and healthy controls. MOG-IgA, MOG-IgG, and MOG-IgM were measured in serum samples and cerebrospinal fluid (CSF) of patients, who were assessed from September 2012 to April 2022.

**Main Outcomes and Measures:**

Frequency and clinical features of patients who were seropositive for MOG-IgA and double-seronegative for aquaporin 4 (AQP4) IgG and MOG-IgG.

**Results:**

After the exclusion of 5 participants with coexisting AQP4-IgG and MOG-IgA, MOG-IgG, and/or MOG-IgM, 1339 patients and 110 healthy controls were included; the median follow-up time was 39 months (range, 0-227 months). Of included patients with isolated MOG-IgA, 11 of 18 were female (61%), and the median age was 31.5 years (range, 3-76 years). Among patients double-seronegative for AQP4-IgG and MOG-IgG (1126/1339; 84%), isolated MOG-IgA was identified in 3 of 50 patients (6%) with neuromyelitis optica spectrum disorder, 5 of 228 patients (2%) with other CNS demyelinating diseases, and 10 of 848 patients (1%) with multiple sclerosis but in none of the healthy controls (0/110). The most common disease manifestation in patients seropositive for isolated MOG-IgA was myelitis (11/17 [65%]), followed by more frequent brainstem syndrome (7/16 [44%] vs 14/75 [19%], respectively; *P* = .048), and infrequent manifestation of optic neuritis (4/15 [27%] vs 46/73 [63%], respectively; *P* = .02) vs patients with MOG-IgG. Among patients fulfilling 2017 McDonald criteria for multiple sclerosis, MOG-IgA was associated with less frequent CSF-specific oligoclonal bands (4/9 [44%] vs 325/351 [93%], respectively; *P* < .001) vs patients with multiple sclerosis who were MOG-IgG/IgA seronegative. Further, most patients with isolated MOG-IgA presented clinical attacks after recent infection or vaccination (7/11 [64%]).

**Conclusion and Relevance:**

In this study, MOG-specific IgA was identified in a subgroup of patients who were double-seronegative for AQP4-/MOG-IgG, suggesting that MOG-IgA may be a novel diagnostic biomarker for patients with CNS demyelination.

## Key Points

**Question:**

What is the frequency of immunoglobulin (Ig) A antibodies against myelin oligodendrocyte glycoprotein (MOG) in patients with central nervous system (CNS) demyelination, and do these antibodies associate with a distinct clinical phenotype?

**Findings:**

In this longitudinal study, a subgroup of patients with demyelinating disorders was double-seronegative for aquaporin 4 (AQP4) IgG and MOG-IgG but seropositive for MOG-IgA. These patients presented with frequent myelitis and brainstem syndrome, infrequent optic nerve involvement, and a low percentage of cerebrospinal fluid–specific oligoclonal band positivity.

**Meaning:**

The findings suggest that MOG-IgA may be a novel diagnostic biomarker in a distinct subgroup of AQP4-/MOG-IgG double-seronegative patients with CNS demyelination.

## Teaser

This cross-sectional study examines laboratory and imaging data for patients with demyelinating central nervous system disease to investigate the frequency of MOG-IgA and associated clinical features.

## Introduction

The identification of aquaporin 4 (AQP4) and myelin oligodendrocyte glycoprotein (MOG) immunoglobulin G (IgG) along with the description of their disease entities^1,2,3,4,5^ has paved the way for serological diagnoses in patients with central nervous system (CNS) demyelination,^6^ including neuromyelitis optica spectrum disorder (NMOSD)^6,7^ and MOG antibody–associated disease.^4,8^ Yet the differential diagnosis and management of patients with AQP4-/MOG-IgG double-seronegative disease remains a challenge.

Recent evidence suggests that IgA may play a role in the pathogenesis of inflammatory disorders.^9,10^ However, the role of autoreactive IgA antibodies in CNS demyelination is still unclear. Here, we conducted an observational, retrospective, longitudinal multicenter study to investigate the frequency of MOG-IgA and its association with clinical features in demyelinating CNS syndromes.

## Methods

### Study Participants

We cross-sectionally screened serum samples from 1344 patients with suspected or confirmed multiple sclerosis (MS),^11^ MOG antibody–associated disease,^8^ or NMOSD^7^ at sampling and 110 healthy controls from 5 centers in a discovery and confirmation setup. Patients were assessed from September 2012 to April 2022 (median follow-up time, 39 months; range, 0-227 months). Both CSF and longitudinal serum samples were measured when available. Five patients were excluded from the study (eMethods in Supplement 1). This study was approved by the institutional review boards of the participating centers. All patients provided written informed consent.

### Clinical and Imaging Data

Retrieval and analysis of available clinical and other data, magnetic resonance images, and retinal optical coherence tomography are described in the eMethods and eTable 1 in Supplement 1.

### Live Cell-Based MOG Assay

Serum samples (1:100) and CSF (1:5) were examined for IgA/IgG/IgM reactivity against full-length human MOG using a live cell-based assay as previously described^3,5^ (eMethods in Supplement 1). For each sample, the ratio of the geometric mean channel fluorescence intensity of the human MOG-transfected cell line divided by the geometric mean channel fluorescence intensity of the control cell line was calculated. Geometric mean channel fluorescence ratio cutoffs were set to 3 SDs and a 25% surplus above the mean values for the healthy controls of the discovery cohort (IgA ≥2.4, IgG ≥3, IgM ≥1.6).

### Statistical Analysis

We used χ^2^ and Fisher exact tests for categorical variables. For continuous variables, we used unpaired *t* tests. The significance cutoff was set at *P* < .05. For optical coherence tomography analyses, we performed linear mixed models at eye level with correction for age and sex (fixed effects) to account for intraparticipant, intereye dependencies. We used Prism 9 version 9.4.1 or R version 4.1.3 (packages: ellipsis, pastecs, readxl, ggplot2, car, lmerTest, MuMIn, Matrix, carData and lme4). Further details are described in the eMethods in Supplement 1.

## Results

**Figure 1.**
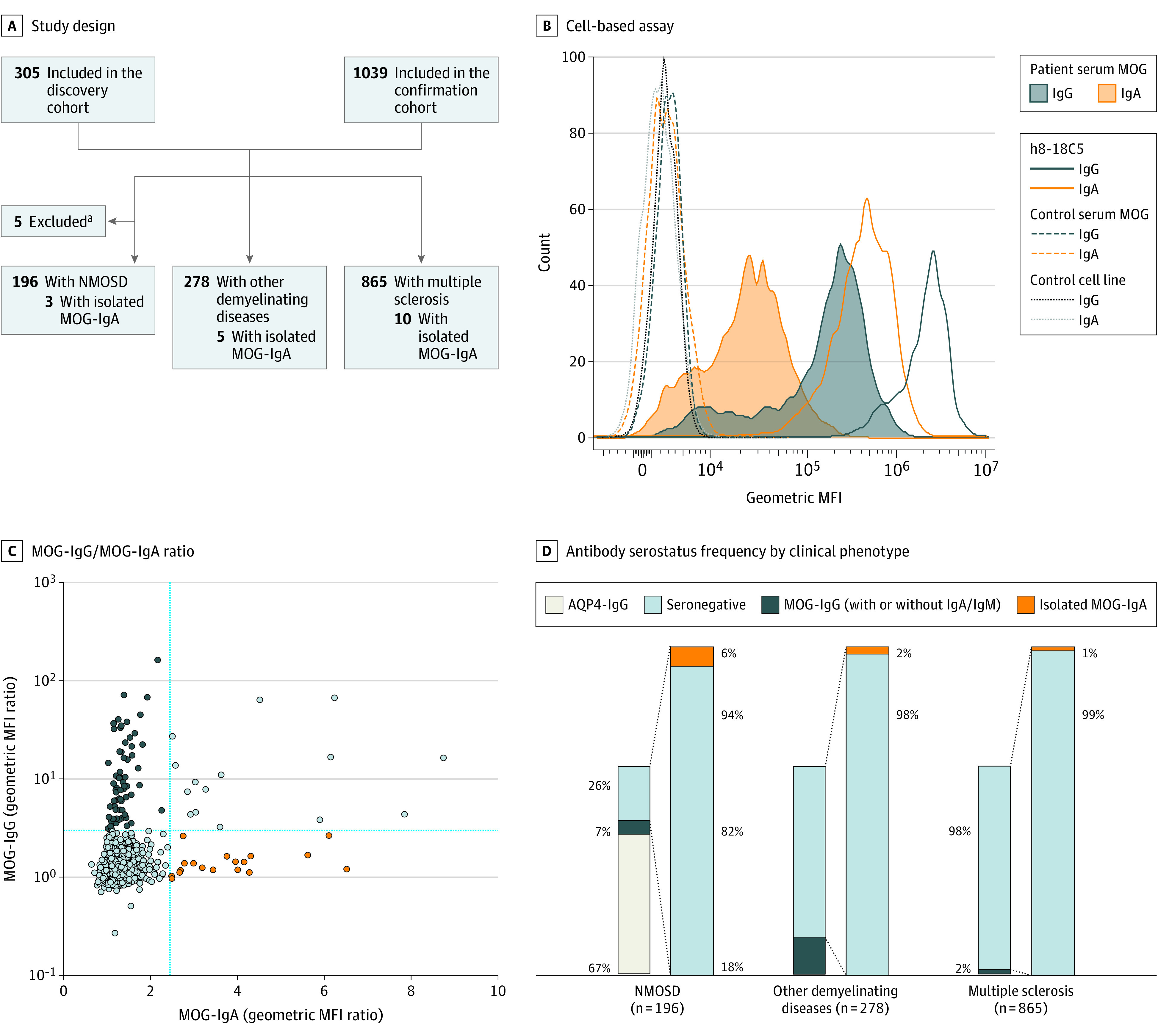
Study Design and Frequency of Isolated Myelin Oligodendrocyte Glycoprotein (MOG) Immunoglobulin (Ig) A in Central Nervous System Demyelination A, Flowchart of patients in the discovery and confirmation cohort who were screened for MOG-IgA, MOG-IgG, and MOG-IgM. Aquaporin 4 (AQP4) was tested as part of the routine clinical diagnosis. B, Representative IgG and IgA binding of humanized 8-18C5 (h8-18C5) monoclonal antibody, MOG-Ig seropositive patient serum, and MOG-Ig seronegative control sample to human MOG–transfected or control cells. C, Individual patients’ geometric mean fluorescence intensity (MFI) ratio based on up to 4 measurements as XY plot for MOG-IgG and MOG-IgA, all cohorts combined. D, Antibody serostatus frequency according to clinical phenotype. Seronegative indicates AQP4-/MOG-IgG double-seronegative. ^a^Five patients with neuromyelitis optica spectrum disorder (NMOSD) were excluded from downstream analysis: 3 with AQP4-IgG/MOG-IgG, 1 with AQP4-IgG/MOG-IgA, and 1 with AQP4-IgG/MOG-IgG/MOG-IgM.

To assess the frequency of MOG-IgA seropositivity, we investigated MOG-IgA, MOG-IgG, and MOG-IgM in 1339 patients with CNS demyelination (MS, n = 865; NMOSD, n = 196; other demyelinating diseases, n = 278) (Figure 1A-C). Overall, MOG-IgG was present in 81 of 1339 patients (6%) (Figure 1C) of whom 18 of 81 (22%) presented either coexisting MOG-IgA (15/81 [19%]) or MOG-IgM (3/81 [14%]) (eFigure 1 and eTable 2 in Supplement 1). Isolated MOG-IgM was identified in 6 additional patients, and 1 patient presented with coexisting MOG-IgM and MOG-IgA. Isolated serum MOG-IgA was identified in 18 of 1126 patients (1.6%) who were double-seronegative for AQP4-/MOG-IgG (Figure 1C and eFigure 1 in Supplement 1) but in none of the available CSF samples (n = 25) or serum samples from controls (n = 110) (eFigure 1 in Supplement 1). MOG-IgA assay specificity was confirmed at 1:20 serum dilution (eFigure 1 in Supplement 1). Demographic and clinical features of patients with isolated MOG-IgA and MOG-IgG are summarized in the Table and eTables 2 and 3 in Supplement 1.

**Table.** Demographic and Clinical Features of Patients Who Were Seropositive for MOG-IgA and MOG-IgG

Characteristic	No./total No. (%)^a^	
	Isolated MOG-IgA (n = 18)	MOG-IgG ± IgA/IgM (n = 81)^b^
Sex		
Female	11 (61)	40 (49)
Male	7 (39)	41 (51)
Age at disease onset, median (range), y	32.5 (3-76)	34 (3-68)
EDSS score at sampling, median (range)	2.75 (0-9.5)	2 (0-8.5)
No. of attacks at last follow-up, median (range)	2 (1-4)	2 (1-14)
Duration of follow-up, median (range), mo	25 (0-108)	43 (0-227)
CSF-specific OCBs	5/16 (31)	16/48 (33)
Untreated patients	7/16 (44)	14/69 (20)

Abbreviations: EDSS, Expanded Disability Status Scale; Ig, immunoglobulin; MOG, myelin oligodendrocyte glycoprotein; OCBs, oligoclonal bands; CSF, cerebrospinal fluid.

^a^ All *P* values for comparisons of characteristics between groups were nonsignificant.

^b^ Patients who were seropositive for MOG-IgG regardless of coexistence of MOG-IgA and/or MOG-IgM.

**Figure 2.**
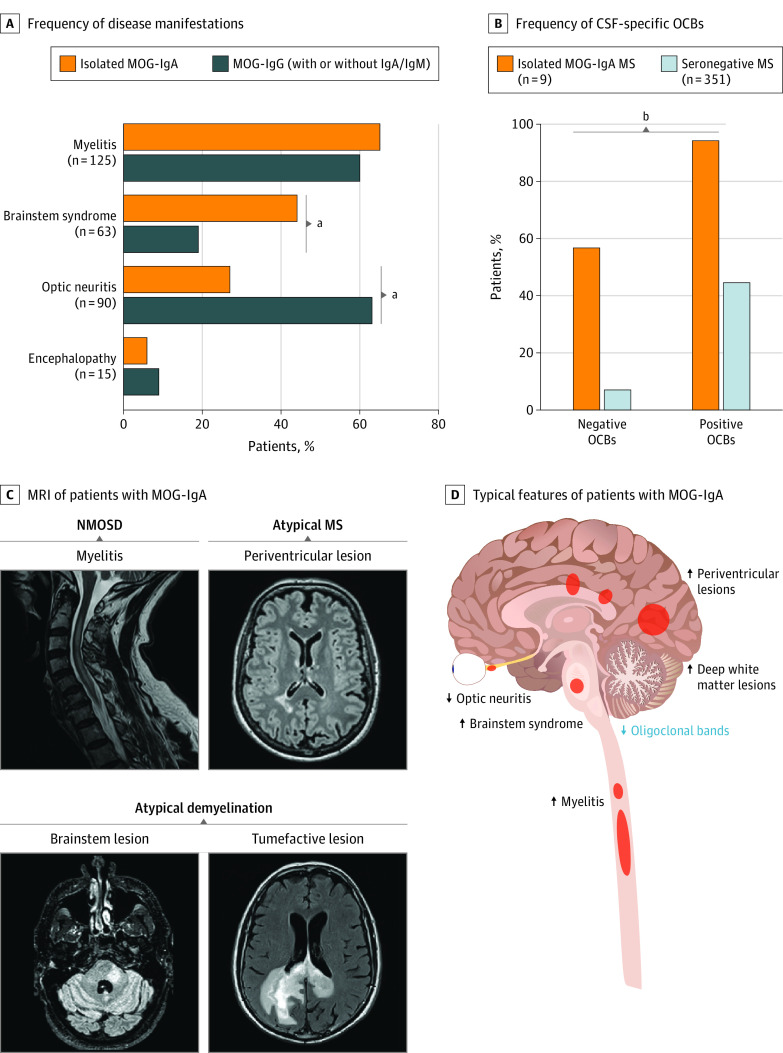
Clinical Characterization of Patients Seropositive for Myelin Oligodendrocyte Glycoprotein (MOG) Immunoglobulin (Ig) A A, Frequency of disease manifestations for patients with isolated MOG-IgA and MOG-IgG. B, Frequency of positive and negative cerebrospinal fluid (CSF)–specific oligoclonal bands (OCBs) in MOG-IgA seropositive multiple sclerosis (MS) compared with seronegative MS. C, Magnetic resonance imaging (MRI) of patients with MOG-IgA highlighting the following disease phenotypes: neuromyelitis optica spectrum disorder (NMOSD, often presenting with myelitis), atypical MS (often presenting with periventricular lesions), and atypical demyelination (often associated with brainstem syndrome or with tumor-mimic/atypical demyelination). D, Clinical features frequently observed in isolated MOG-IgA seropositive central nervous system demyelination. Arrows indicate high and low frequencies. ^a^Fisher exact test, *P* < .05. ^b^Fisher exact test, *P* < .001.

MOG-IgA was positive in 3 of 50 patients (6%) with NMOSD, in 5 of 228 patients (2%) with other demyelinating diseases, and in 10 of 848 patients (1%) with MS who were double-seronegative for AQP4-/MOG-IgG (Figure 1D). Myelitis (11/17 [65%]) was the most frequent disease manifestation, followed by brainstem syndrome (7/16 [44%] vs 14/75 [19%], respectively; *P* = .048), which occurred at a higher frequency than in patients with MOG-IgG. Optic neuritis was less frequent in the isolated MOG-IgA group (4/15 [27%] vs 46/73 [63%] in the MOG-IgG group; *P* = .02) (Figure 2A and eFigure 2 in Supplement 1). Peripapillary retinal nerve fiber layer and ganglion cell–inner plexiform layer thicknesses in eyes of patients with isolated MOG-IgA and optic neuritis were not different from those of MOG-IgG patients with optic neuritis (eFigure 3 in Supplement 1). Additionally, no significant differences in the frequency of disease manifestations were detected in other MOG-Ig isotype groups (MOG-IgM, MOG-IgG/A, MOG-IgG/M), except for a difference in optic neuritis frequency comparing isolated MOG-IgA with isolated MOG-IgG (35/55 [64%]) (eFigure 2 in Supplement 1).

Interestingly, only 4 of 9 patients (44%) who were seropositive for isolated MOG-IgA and had a diagnosis of MS^11^ presented CSF-specific OCBs, clearly less than in those with MOG-IgA/-IgG seronegative MS (4/9 [44%] vs 325/351 [93%], respectively; *P* < .001) (Figure 2B and eTable 3 in Supplement 1). Overall, patients with isolated MOG-IgA presented at least 1 of the following imaging features: (1) myelitis (short or longitudinally extensive); (2) periventricular lesion; (3) tumefactive deep white matter lesion; and (4) brainstem lesion, resembling NMOSD, atypical MS, and atypical demyelination phenotypes (Figure 2C and D and eFigure 4 in Supplement 1).

Investigating the frequency of patients with records of clinical attacks (onset or relapses) reported within 3 months following infection or vaccination, we observed no significant difference between the isolated MOG-IgA (7/11 [64%]) and MOG-IgG (7/19 [37%]) groups. No association with specific vaccines or pathogens was observed (eTable 3 in Supplement 1). Furthermore, there was no evidence of seroconversion from neither MOG-IgM/-IgG nor MOG-Ig seronegative to MOG-IgA in patients with available longitudinal samples (n = 90) (eMethods and eFigure 5 in Supplement 1).

## Discussion

We identified isolated MOG-IgA in a small subset of patients presenting with myelitis, brainstem syndrome, and infrequent optic neuritis overlapping with core clinical features of NMOSD^7^ and MOG antibody–associated disease.^8^ While the coexistence of MOG-IgM and MOG-IgA has previously been described^12^ in a similar frequency as detected in our cohort, we expand on the existing literature by reporting isolated MOG-IgA seropositivity in patients seronegative for MOG-IgG/-IgM and AQP4-IgG.

Unlike IgG, which is mounted systemically, IgA is mainly produced in mucosal tissues where it serves as a first-line barrier against pathogens and commensals, raising questions about the different mechanisms of immune activation that lead to divergent MOG-Ig responses. Although a high frequency of patients who were seropositive for isolated MOG-IgA showed records of attacks preceded by infections or vaccinations, we did not observe associations with specific triggers. An alternative explanation for the occurrence of isolated MOG-IgA could be subsequent seroconversion from MOG-IgM or MOG-IgG induced by the inflammatory milieu. While our longitudinal data of unchanged MOG-Ig isotype patterns over time argue against this, little is known about disease-specific induction of isolated IgA responses.^9^ Future studies are required to investigate the clinical relevance of both isolated and coexisting MOG-IgG/-IgA seropositivity.

In contrast to IgG, which is known for its proinflammatory role through complement activation,^4,6^ the pathogenic potential of IgA is debated.^9^ Yet evidence suggests that IgA may target neuronal and myelin antigens^13,14^ in CNS inflammation, and a proinflammatory role via IgA immune complex formation and subsequent immune activation has been described in several diseases.^9^ The distinct clinical syndrome in patients seropositive for isolated MOG-IgA, characterized by frequent inflammation of the brainstem and spinal cord, areas with high blood-brain barrier permeability,^15^ further suggests that IgA may have a pathogenic role in CNS inflammation. Prospective studies investigating immune activation mechanisms and transferring MOG-IgA into animals will be important steps to assess pathogenicity and clarify the etiology of MOG-IgA–associated disease.

### Limitations

Our study has several limitations. First, the clinical data were mostly obtained retrospectively with some unavailable clinical variables; therefore, we cannot exclude the possibility of recollection bias. Second, serum samples were not always collected from untreated patients, possibly underestimating the detected frequency of MOG-IgA/-IgG/-IgM. Further, the small number of patients seropositive for isolated MOG-IgA may have underpowered the detection of additional clinical and other differences, compromising the generalizability of the findings.

## Conclusions

In this study, MOG-specific IgA was identified in a subgroup of patients who were double-seronegative for AQP4-/MOG-IgG and presented with distinct clinical features. This finding suggests a potential use of MOG-IgA as a biomarker in AQP4-/MOG-IgG double-seronegative CNS demyelination. Further prospective studies are required to enhance the characterization of the syndrome and decipher underlying pathogenic mechanisms.

## Notes

Supplement 1.
eMethods
**eFigure 1.** Serum and CSF (cerebrospinal fluid) myelin oligodendrocyte glycoprotein (MOG) immunoglobulin (Ig) levels **eFigure 2.** Disease manifestations in MOG-Ig isotype subgroups **eFigure 3.** Optical coherence tomography (OCT) stratified by optic neuritis (ON) **eFigure 4.** Brain and spinal magnetic resonance imaging (MRI) **eFigure 5.** Longitudinal MOG-Ig serolevels **eTable 1.** Non-available data for clinical parameters **eTable 2.** Demographic and clinical features of MOG-IgA and -IgG seropositive patients of the discovery, confirmation, and combined cohorts **eTable 3.** Clinical characteristics of MOG-IgA seropositive patients
eReferences

Supplement 2. Data sharing statement
