# Supplementary material for: Immunoglobulin A Antibodies Against Myelin Oligodendrocyte Glycoprotein in a Subgroup of Patients With Central Nervous System Demyelination
Source: JAMA Neurol. 2023 Aug 7;80(9):989–95. doi: 10.1001/jamaneurol.2023.2523 (PMC10407763; doi:10.1001/jamaneurol.2023.2523)
Supplement: Supplement 1. — eMethods eFigure 1. Serum and CSF (cerebrospinal fluid) myelin oligodendrocyte glycoprotein (MOG) immunoglobulin (Ig) levels eFigure 2. Disease manifestations in MOG-Ig isotype subgroups eFigure 3. Optical coherence tomography (OCT) stratified by optic neuritis (ON) eFigure 4. Brain and spinal magnetic resonance imaging (MRI) eFigure 5. Longitudinal MOG-Ig serolevels eTable 1. Non-available data for clinical parameters eTable 2. Demographic and clinical features of MOG-IgA and -IgG seropositive patients of the discovery, confirmation, and combined cohorts eTable 3. Clinical characteristics of MOG-IgA seropositive patients eReferences [file jamaneurol-e232523-s001.pdf]

Supplemental Online Content

Ayroza Galvão Ribeiro Gomes AB, Kulsvehagen L, Lipps P, et al. Immunoglobulin A antibodies against myelin oligodendrocyte glycoprotein in a subgroup of patients with central nervous system demyelination. *JAMA Neurol*. Published online August 7, 2023. doi:10.1001/jamaneurol.2023.2523

eMethods

eFigure 1. Serum and CSF (cerebrospinal fluid) myelin oligodendrocyte glycoprotein (MOG) immunoglobulin (Ig) levels

eFigure 2. Disease manifestations in MOG-Ig isotype subgroups

eFigure 3. Optical coherence tomography (OCT) stratified by optic neuritis (ON)

eFigure 4. Brain and spinal magnetic resonance imaging (MRI)

eFigure 5. Longitudinal MOG-Ig serolevels

eTable 1. Non-available data for clinical parameters

eTable 2. Demographic and clinical features of MOG-IgA and -IgG seropositive patients of the discovery, confirmation, and combined cohorts

eTable 3. Clinical characteristics of MOG-IgA seropositive patients

eReferences

This supplemental material has been provided by the authors to give readers additional information about their work.

© 2023 Ayroza Galvão Ribeiro Gomes AB et al. *JAMA Neurology.*

eMethods

Study participants

We cross-sectionally screened sera from 305 adult patients with medical records reporting suspected (isolated optic neuritis/transverse myelitis [ON/TM], acute disseminated encephalomyelitis [ADEM], clinically isolated syndrome [CIS], or radiologically isolated syndrome [RIS]), or confirmed multiple sclerosis (MS)[11], myelin oligodendrocyte glycoprotein (MOG) antibody-associated disease (MOGAD)[8], or neuromyelitis optica spectrum disorder (NMOSD)[6] assessed at the University Hospital of Basel between September 2012 and April 2022. Additionally, sera from 39 healthy controls (HC) were screened.

Findings of the discovery cohort prompted us to procure additional 1039 cross-sectional samples from other independent cohorts (confirmation cohort): n=698, Swiss MS Cohort Study (center Basel), Switzerland[16]; n=187, Hospital das Clínicas HCFMUSP, São Paulo, Brazil; n=76, Charité-Universitätsmedizin Berlin, Germany; n=39, St. Josef-Hospital, Ruhr University Bochum, Germany; n=39, Heinrich Heine University Düsseldorf, Germany. In addition, 71 HC from two centers were recruited as part of the confirmation cohort. Longitudinal samples were measured when available for MOG-Ig seropositive patients (n=48) and MOG-Ig seronegative patients (n=42). A median of 3 (range 2-17) samples were tested per patient over a median of 28.7 (range 0.2-116.4) months. Patients with co-existing AQP4-IgG and MOG-IgG/-M/-A antibodies (n=5/1344) had AQP4-IgG remeasured by an independent laboratory (Laboratory Krone, Bad Salzuflen, Germany) to confirm its positivity and, upon confirmation, were excluded from all analyses to prevent analytic confounding between MOG-Ig isotypes and clinical features, as data suggests that patients with coexisting AQP4-IgG and MOG-IgG manifest with a clinical course consistent with AQP4-IgG seropositive NMOSD[17].

Clinical and laboratory data

Available clinical data and diagnostic AQP4-antibody results were retrieved from hospital records, research databases, or electronic surveys. Clinical manifestations (brainstem

© 2023 Ayroza Galvão Ribeiro Gomes AB et al. *JAMA Neurology.*

syndrome, encephalopathy, ON, myelitis) were described as topographic syndromes consisting of signs and symptoms confirmed by magnetic resonance imaging (MRI), retinal optical coherence tomography (OCT), or electrophysiological findings presented during attacks. Clinical phenotypes were categorized into MS (fulfillment of McDonald 2017 criteria for diagnosis of MS[11]), NMOSD (fulfillment of 2015 international consensus diagnostic criteria for diagnosis of NMOSD[6]), and other demyelinating diseases (patients not categorized as MS or NMOSD). Missing data is referred to as non-available (**eTable 1**). Vaccination and infection history was obtained via electronic survey and medical record/laboratory data review when available (n=30). EQUATOR (STROBE) reporting guidelines were followed.

Imaging data

Analysis of clinically available magnetic resonance images (MRI) was performed by consensus by three raters (A.C., R.C., N.S.) blinded to the clinical and serological data. Brain T2-hyperintense lesions were classified as periventricular, juxtacortical, deep white matter, deep gray matter, brainstem, and cerebellar[18]. Cortical lesions were identified on magnetization-prepared rapid gradient-echo (MPRAGE) images and categorized as either intracortical or leukocortical. The number, location, and longitudinal extension of spinal cord lesions were assessed on sagittal and axial T2-turbo spin echo (T2-TSE) images.

All OCTs were performed on Heidelberg Engineering Spectralis devices (Heidelberg, Germany). We report the OCT-acquisition settings and scanning according to a previously described protocol[19]. All images underwent the quality assessment according to the OSCAR-IB criteria[20]. The peripapillary retinal nerve fiber layer (pRNFL) was measured using 3.5-mm ring scans around the optic nerve head (12°, 1536 A-scans, 9≤ ART ≤100). The mean thickness was used in the analysis. Ring scans of three centers were measured with the “standard” Spectralis protocol, but two centers provided measurements using an anatomic positioning system (APS-RNFL). For the latter, we used the mean pRNFL thickness of the 3.4 mm ring. Due to the overall small number of eyes with MOG-IgA, we analyzed all ring scans

© 2023 Ayroza Galvão Ribeiro Gomes AB et al. *JAMA Neurology.*

(standard and APS) together. The volume of macular layers was provided from volume scans (25° x 30°, 61 vertical B-scans, 768 A-scans per B-scan, 11 ≤ ART ≤22). After an initial automated segmentation of all retinal layers using the software provided by Heidelberg Engineering (Eye Explorer 1.9.13.0), segmentation results were checked and corrected when needed by one experienced rater (NCF). The final segmentation was approved by a second experienced rater (AP). For the analysis, the volume of the inner nuclear layer (INL) and the combined GCIPL volume (composite of ganglion cell layer, GCL and inner plexiform layer, IPL, volumes) was used, in the 3 mm-diameter cylinder of the 1,2,3 mm ring adjacent to the fovea. Of 61 patients with isolated MOG-IgA or MOG-IgG for whom the OCT was available, 3 patients (2 eyes per patient) were excluded (n=1, unclear history of optic neuritis; n=2, incidental findings). Additionally, 7 eyes (one per patient) were excluded (n=2, incidental findings; n=5 eyes, unavailable OCT). We included 109 eyes from 58 patients in the analysis, of which 36 maculas were excluded (n=34, different protocols; n=2, incidental findings). Thus, a total of 109 ring scans (n= 17 for MOG-IgA and n= 92 for MOG-IgG) and 73 macular volume scans (n= 13 for MOG-IgA and n= 60 for MOG-IgG) were included in the analysis. The time between scans and ON was the same between the compared groups (isolated MOG-IgA, median of 20 months; MOG-IgG, median of 58 months; p=0.16).

Live cell-based MOG assay

The human rhabdomyosarcoma cell line TE 671 stably transfected with the pRSV neo plasmid containing the full-length sequence of human MOG (247 amino acids, MOG cell line) or the empty vector (control cell line) was used to perform the live cell-based assay as previously described[3,5]. Humanized monoclonal 8-18C5 (h8-18C5)[21] of IgG or IgA isotypes were included as positive controls. Secondary antibodies (all Jackson ImmunoResearch) against human IgA (α chain specific, 1:400, 109-605-011), IgG (Fcγ fragment specific, 1:800, 109-116-098), and IgM (Fc5μ fragment specific, 1:400, 109-545-129) were used to detect surface bound MOG-specific antibodies. MOG-IgA positive sera were repeated at 1:20 and 1:100 dilutions to confirm assay specificity.

© 2023 Ayroza Galvão Ribeiro Gomes AB et al. *JAMA Neurology.*

Statistical analysis

Following the current clinical gold standard of diagnosing MOGAD based on positive MOG-IgG regardless of co-existing -IgM and/or -IgA, the main comparator that we used in the manuscript was isolated MOG-IgA (MOG-$IgA^{Isolated}$) versus MOG-IgG regardless of co-existing -IgM and/or -IgA (MOG-$IgG^{\pm IgA/M}$) unless otherwise stated. For the unadjusted comparison of clinical, oligoclonal bands (OCBs), and MRI data between different antibody groups, we used $\chi 2$ and Fisher exact tests for categorical variables. For continuous variables, we used unpaired t tests. The significance cut-off was set at $p<0.05$. P values were designated as follows: *p≤0.05, **p≤0.01, ***p≤0.001, and ****p≤0.0001. For OCT analyses, we performed linear mixed models (LMM) at eye level with correction for age and sex (fixed effects) to account for intra-subject, inter-eye dependencies. We performed subgroup analysis according to the history of ON corrected for number of episodes.

© 2023 Ayroza Galvão Ribeiro Gomes AB et al. *JAMA Neurology.*

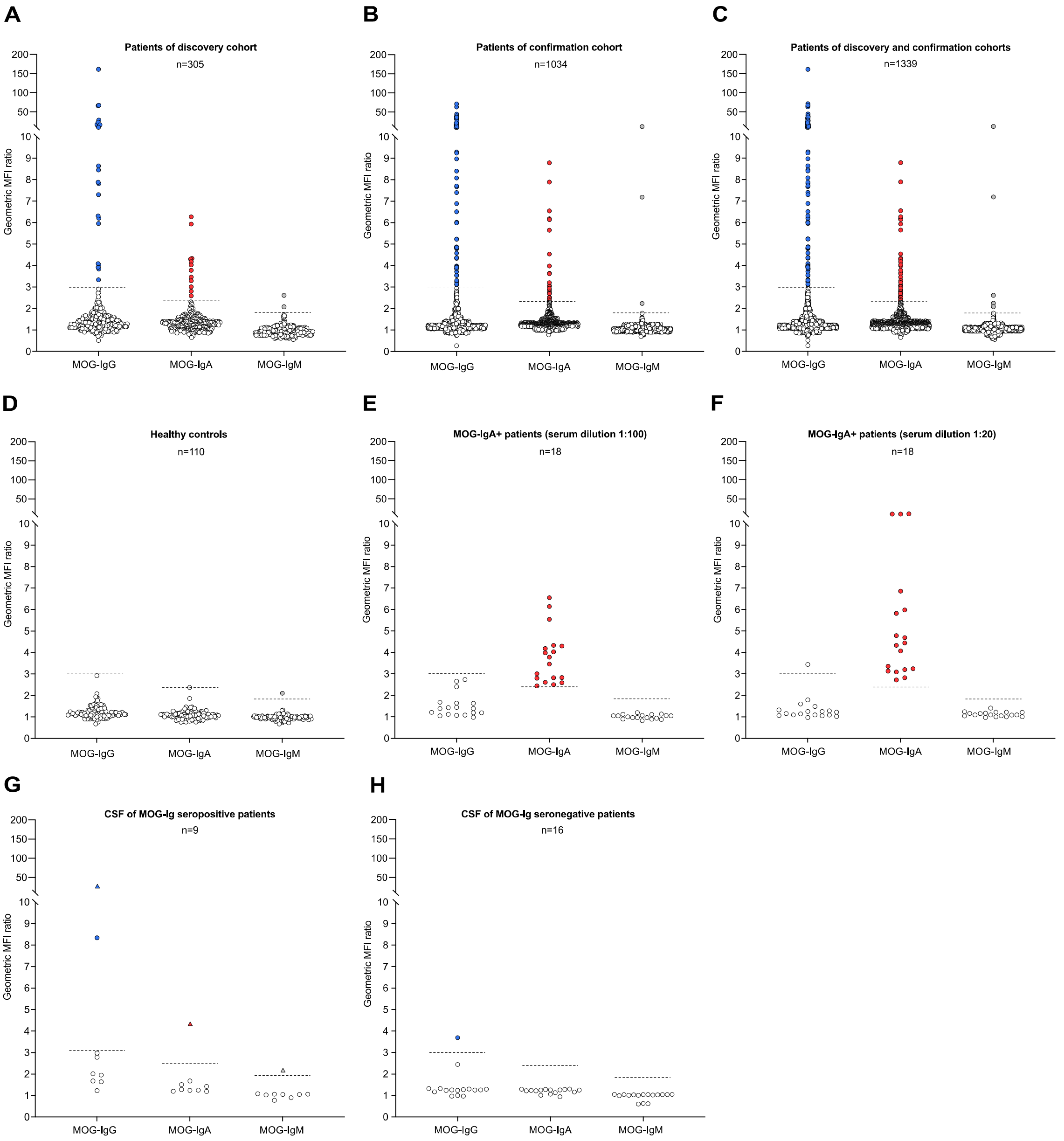

eFigure 1: Serum and CSF (cerebrospinal fluid) myelin oligodendrocyte glycoprotein (MOG) immunoglobulin (Ig) levels. Individual patients' measurements plotted as (average) geometric mean fluorescence intensity (geometric MFI) ratio of up to 4 individual experiments for MOG-IgG, -IgA and -IgM. **A)** Serum of patients of the discovery cohort. **B)** Serum of patients of the confirmation cohort. **C)** Serum of patients of pooled cohorts. **D)** Serum of healthy controls. **E)** Serum of isolated MOG-IgA seropositive patients (n=18) measured at a serum dilution of 1:100. **F)** Serum of isolated MOG-IgA seropositive patients (n=18) measured

© 2023 Ayroza Galvão Ribeiro Gomes AB et al. *JAMA Neurology.*

at a dilution of 1:20. **G)** CSF from available MOG-Ig seropositive patients (n=9) measured at a 1:5 dilution. Triangle indicates MOG-Ig levels from one MOG-IgG seropositive patient with positive MOG-IgG, -IgM, and -IgA levels in CSF. **H)** CSF from available patients seronegative for any MOG-Ig measured at a 1:5 CSF dilution.

© 2023 Ayroza Galvão Ribeiro Gomes AB et al. *JAMA Neurology.*

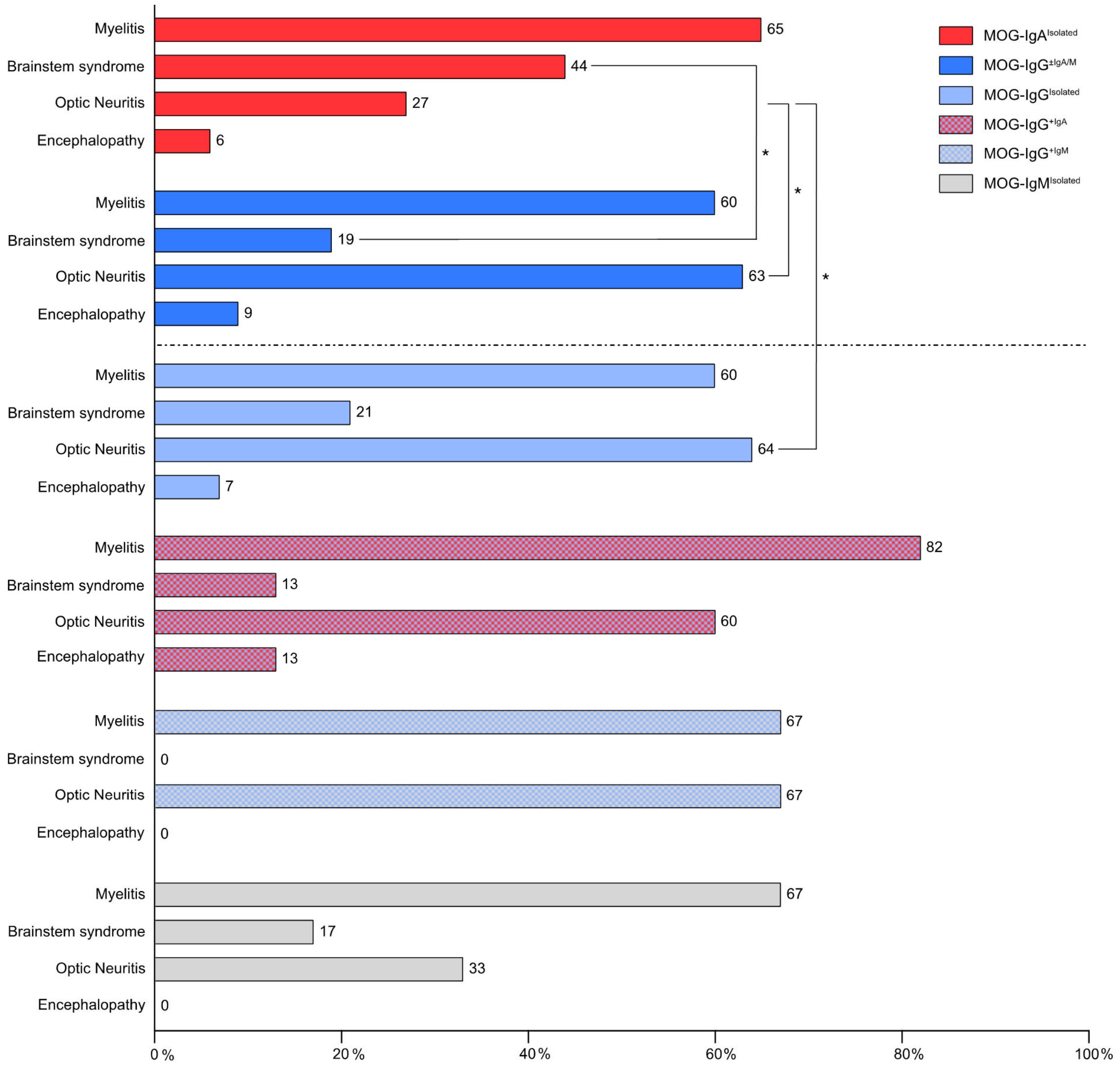

eFigure 2: Disease manifestations in MOG-Ig isotype subgroups. Disease manifestations in patients with MOG-IgA^{Isolated} (red), MOG-IgG$^{\pm IgA/M}$ (MOG-IgG regardless of coexisting MOG-IgA/M serostatus, blue), MOG-IgG^{Isolated} (light blue), MOG-IgG^{+IgA} (double-positive MOG-IgG/A, light blue-red checkered), MOG-IgG^{+IgM} (double-positive MOG-IgG/M, light blue-gray checkered) and MOG-IgM^{Isolated} (gray). Fisher's exact test, *$p<0.05$.

© 2023 Ayroza Galvão Ribeiro Gomes AB et al. *JAMA Neurology.*

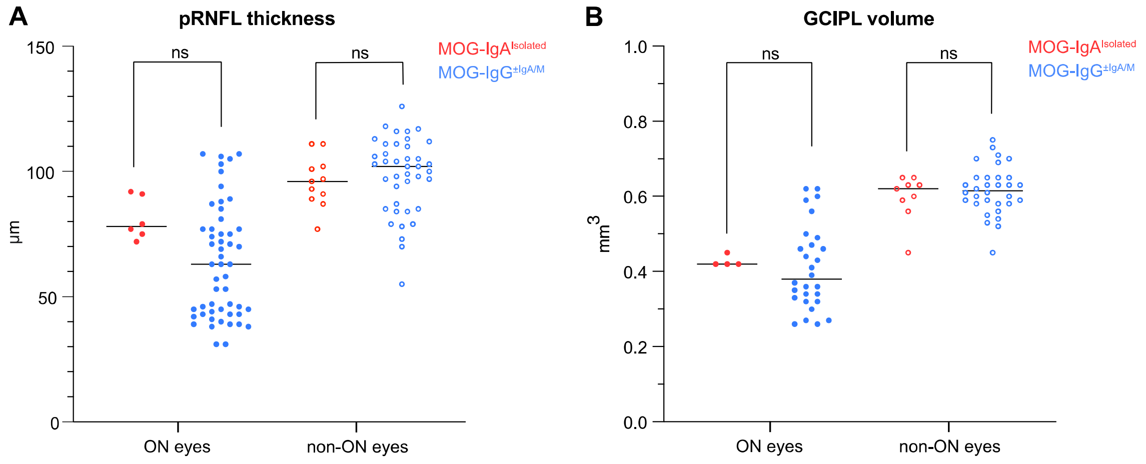

eFigure 3: Optical coherence tomography (OCT) stratified by optic neuritis (ON). A) Mean peripapillary retinal nerve fiber layer (pRNFL) thickness for MOG-IgA^{Isolated} and MOG-IgG$^{\pm IgA/M}$ patients. **B)** Mean ganglion cell-inner plexiform layer (GCIPL) volume for MOG-IgA^{Isolated} and MOG-IgG$^{\pm IgA/M}$ patients. Each data point represents one eye, three out of four isolated MOG-IgA patients included in the analysis had bilateral ON.

© 2023 Ayroza Galvão Ribeiro Gomes AB et al. *JAMA Neurology.*

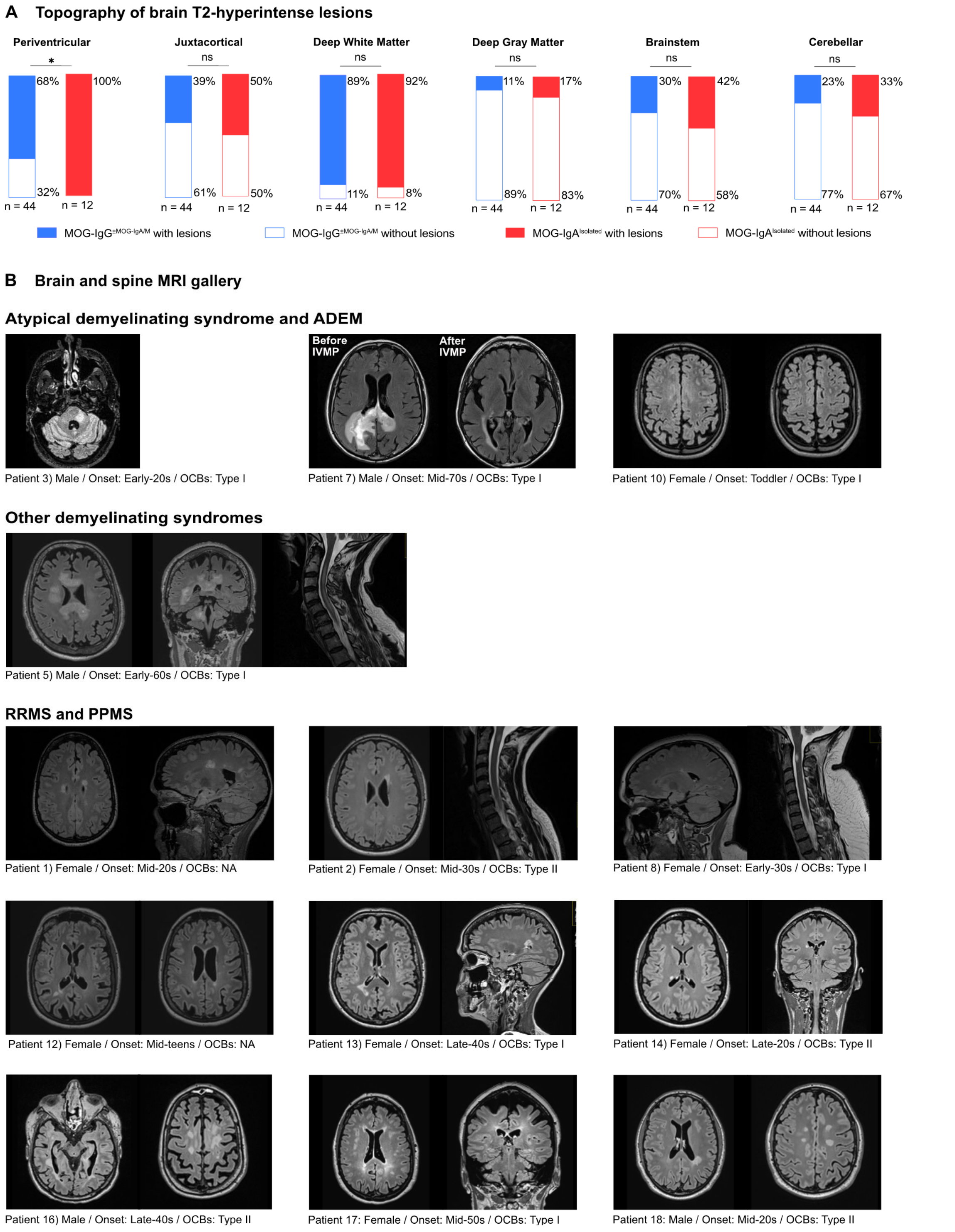

eFigure 4: Brain and spinal magnetic resonance imaging (MRI). A) Topography of brain T2-hyperintense lesions in MOG-IgG and -IgA seropositive patients. Fisher's exact test, *p<0.05. **B)** Image gallery of all available MOG-IgA brain and spine MRIs. Running diagnoses per patient; Patient 1: RRMS, Patient 2: RRMS, Patient 3: Atypical demyelinating syndrome,

© 2023 Ayroza Galvão Ribeiro Gomes AB et al. *JAMA Neurology.*

Patient 5, Seronegative NMOSD and diffuse cerebral B cell lymphoma; Patient 7: Atypical demyelinating syndrome, Patient 8: RRMS, Patient 10: ADEM, Patient 12: RRMS, Patient 13: RRMS; Patient 14: RRMS, Patient 16, PPMS; Patient 17, PPMS; Patient 18, RRMS. ADEM=acute demyelinating encephalomyelitis, RRMS=relapsing-remitting multiple sclerosis (MS), PPMS=primary progressive MS, OCBs=oligoclonal bands, IVMP=Intravenous methylprednisolone.

© 2023 Ayroza Galvão Ribeiro Gomes AB et al. *JAMA Neurology.*

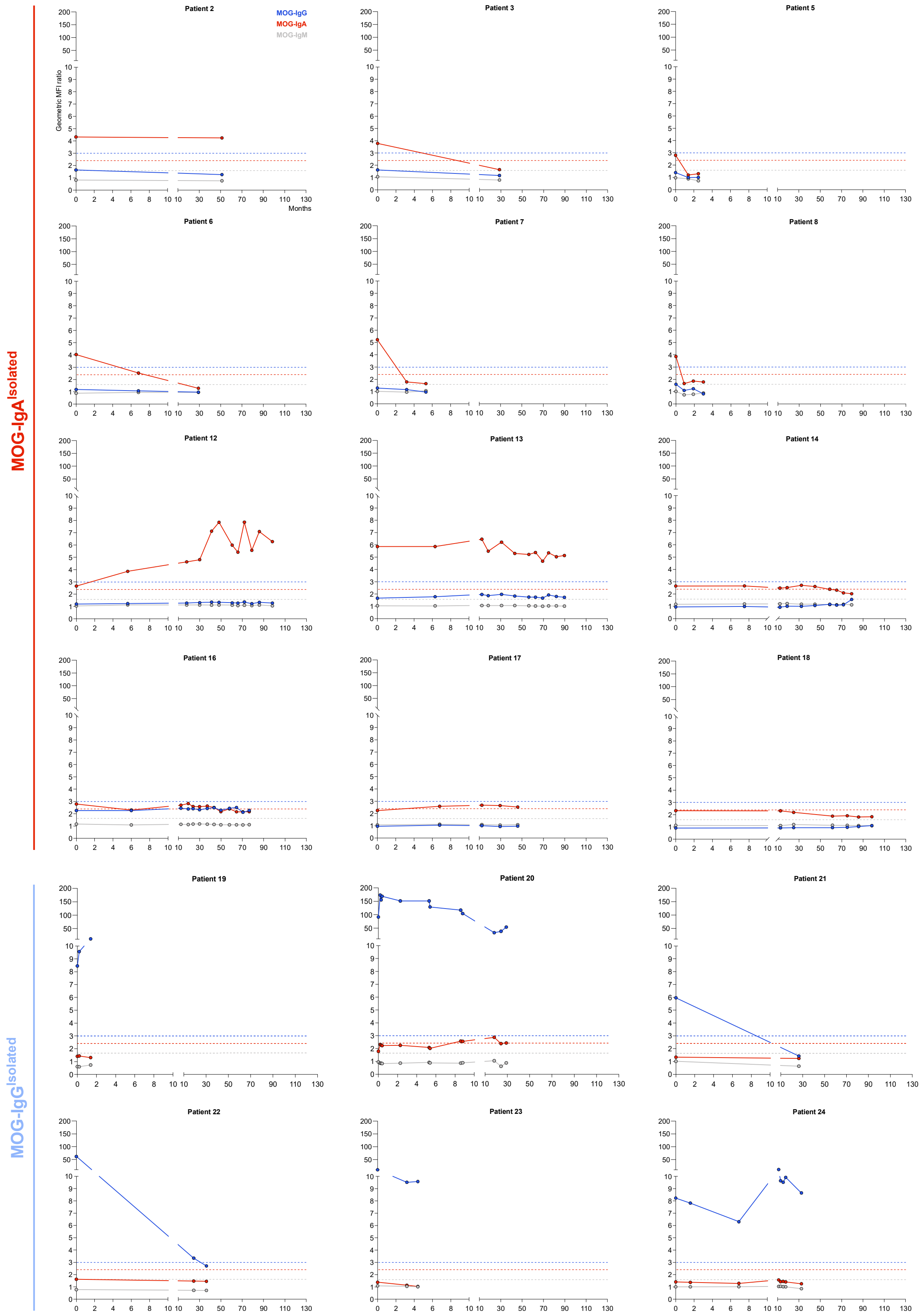

© 2023 Ayroza Galvão Ribeiro Gomes AB et al. *JAMA Neurology.*

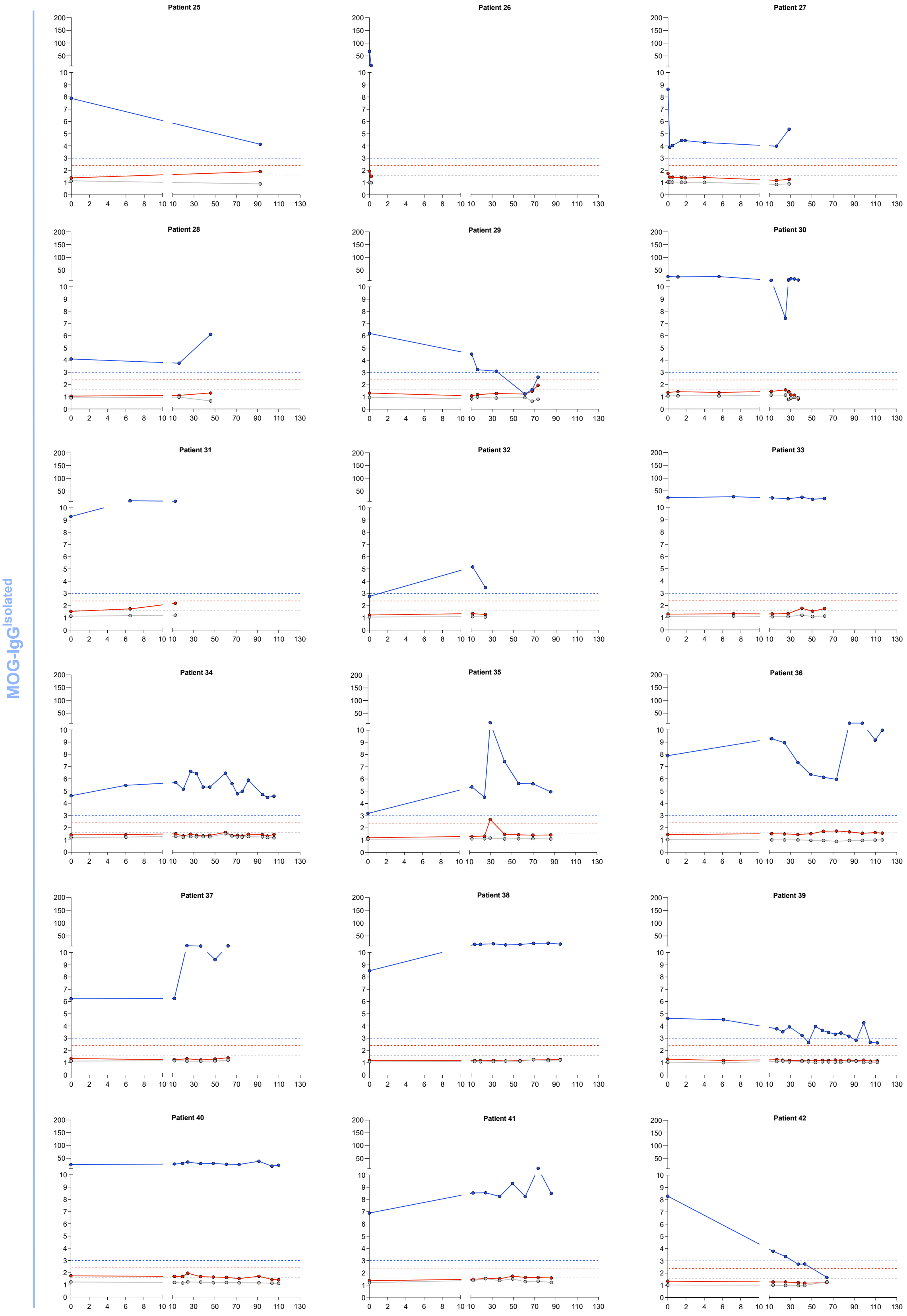

© 2023 Ayroza Galvão Ribeiro Gomes AB et al. *JAMA Neurology.*

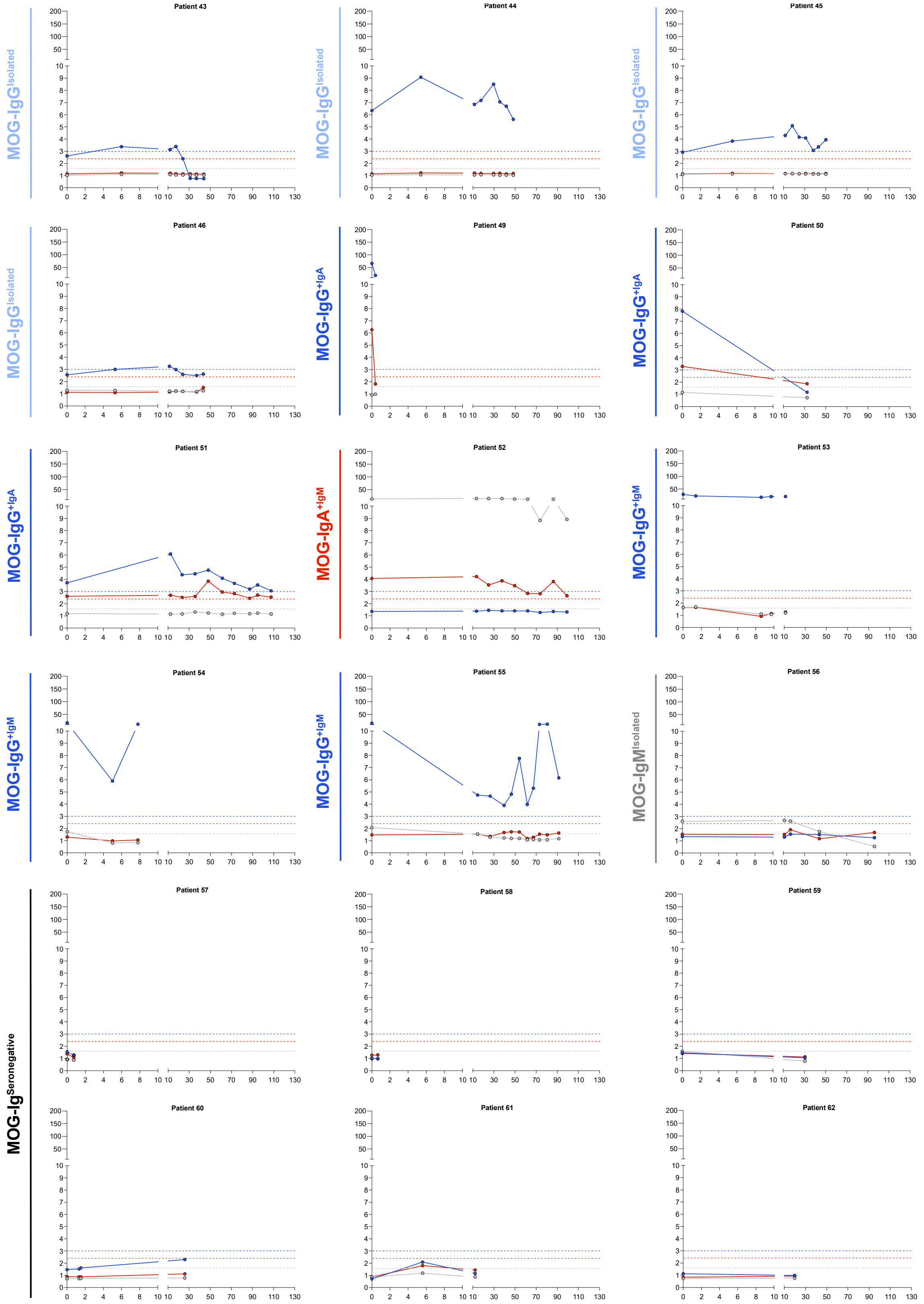

© 2023 Ayroza Galvão Ribeiro Gomes AB et al. *JAMA Neurology.*

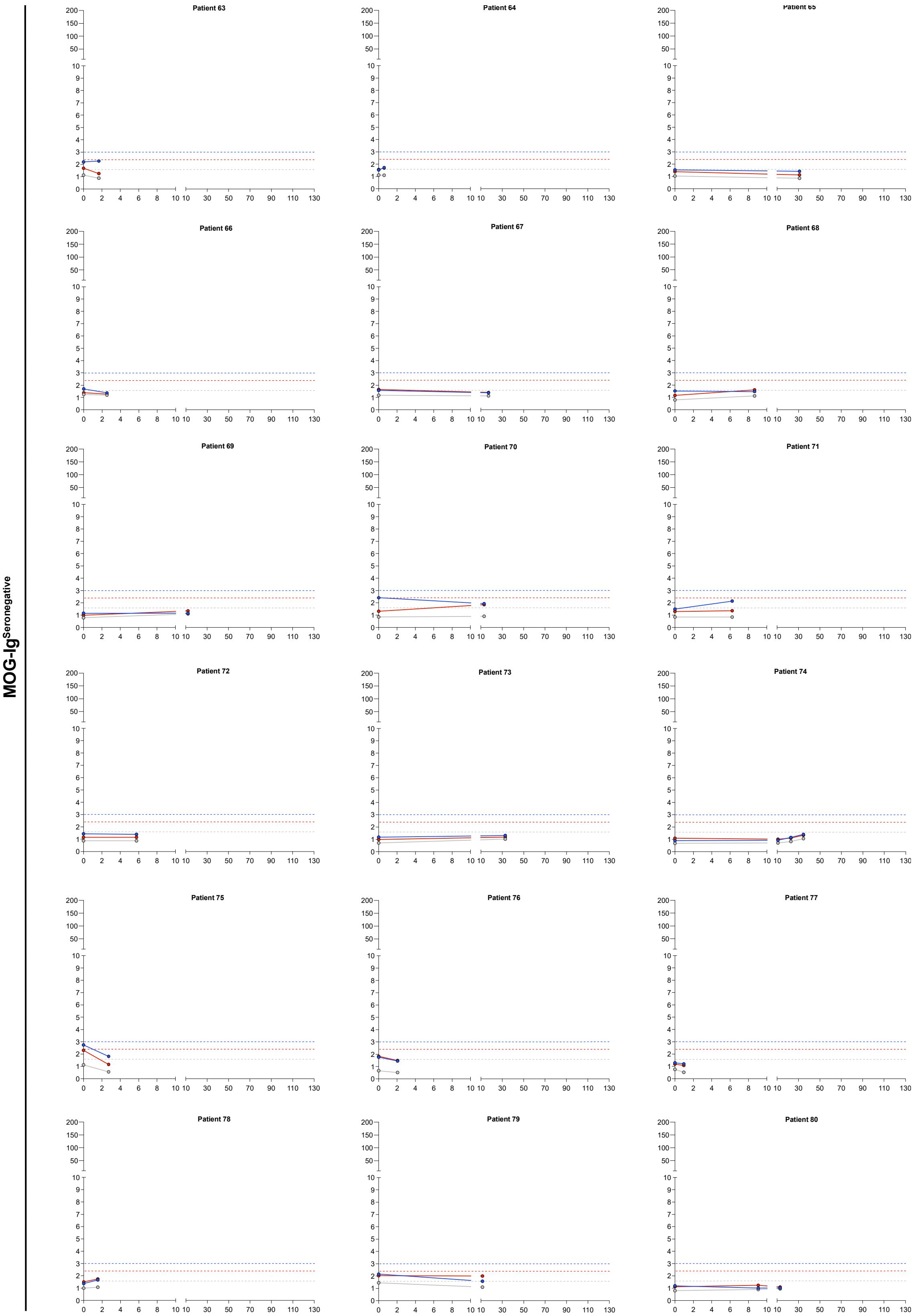

© 2023 Ayroza Galvão Ribeiro Gomes AB et al. *JAMA Neurology.*

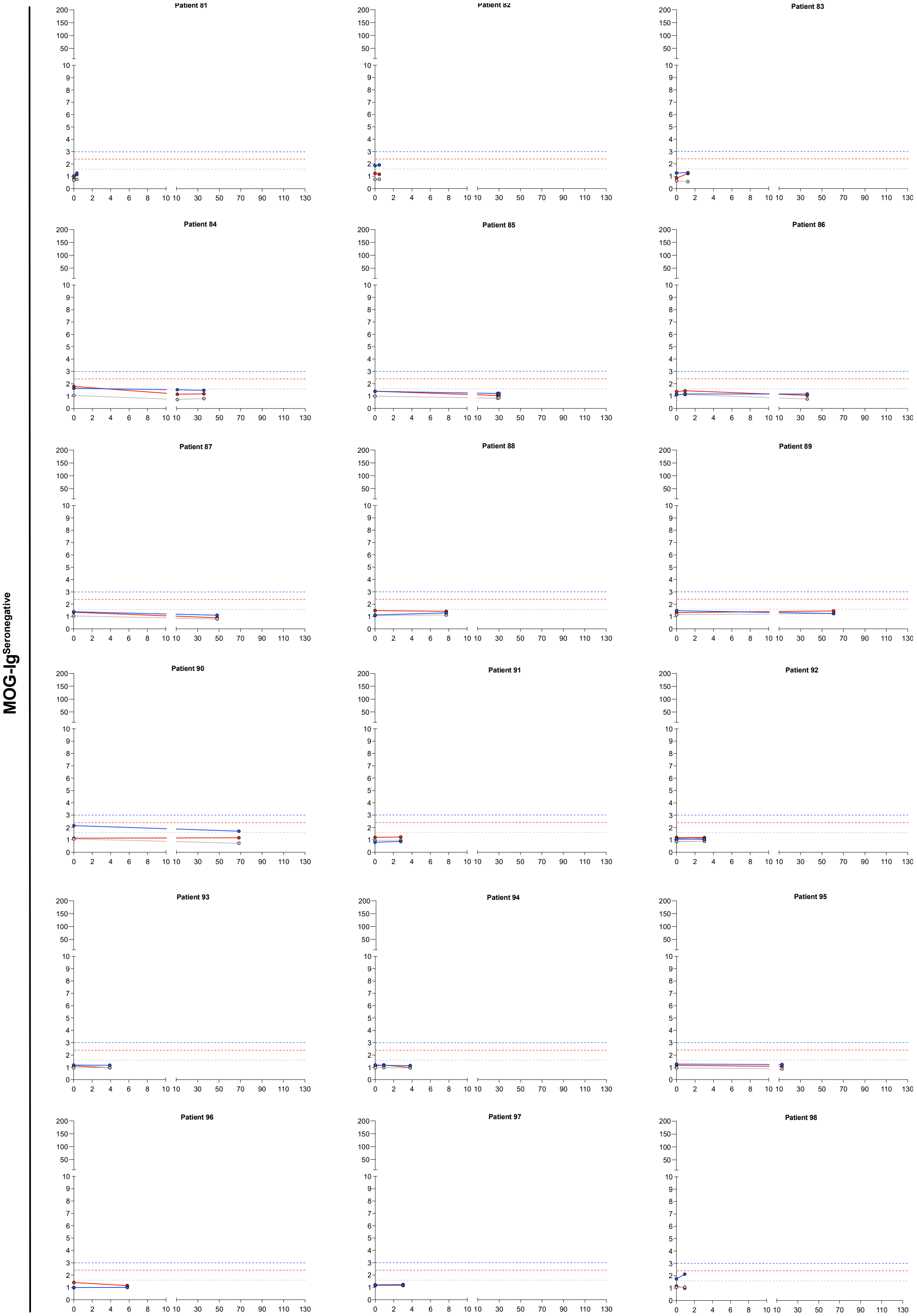

© 2023 Ayroza Galvão Ribeiro Gomes AB et al. *JAMA Neurology.*

eFigure 5: Longitudinal MOG-Ig serolevels. Longitudinally plotted serum levels of MOG-IgA, -IgG, and -IgM for individual patients (n=90; MOG-IgA^{Isolated} patients n=12, MOG-IgG^{Isolated} patients n=28, MOG-IgG^{+IgA} patients n=3, MOG-IgA^{+IgM} patients n=1, MOG-IgG^{+IgM} patients n=3, isolated MOG-IgM^{Isolated} n=1, MOG-Ig^{Seronegative} patients n=42). Dotted lines represent cut-offs for antibody isotypes of respective colors.

© 2023 Ayroza Galvão Ribeiro Gomes AB et al. *JAMA Neurology.*

	MOG-IgA^{Isolated} (n=18)			MOG-IgG$^{±IgA/M}$ (n=81)			MOG-IgA^{Isolated} (n=15) from Basel			MOG-IgG$^{±IgA/M}$ (n=51) from Basel			MOG-IgA^{Isolated} MS (n=10)			MOG-Ig^{Seronegative} MS (n=838)		
	P	A	N.A.	P	A	N.A.	P	A	N.A.	P	A	N.A.	P	A	N.A.	P	A	N.A.
Disease manifestations																		
Myelitis (n)	11	6	1	47	31	3	-	-	-	-	-	-	-	-	-	-	-	-
Brainstem syndrome (n)	7	9	2	14	61	6	-	-	-	-	-	-	-	-	-	-	-	-
ON (n)	4	11	3	46	27	8	-	-	-	-	-	-	-	-	-	-	-	-
Encephalopathy (n)	1	15	2	7	67	7	-	-	-	-	-	-	-	-	-	-	-	-
Paraclinical																		
CSF-specific OCBs	5	11	2	16	32	33	-	-	-	-	-	-	4	5	1	325	26	487
History																		
Preceding vaccination/infection	-	-	-	-	-	-	7	4	4	7	12	32	-	-	-	-	-	-

eTable 1: Non-available data for clinical parameters. MS=multiple sclerosis, P=Present, A=Absent, N.A.=non-available, ON=optic neuritis, CSF=cerebrospinal fluid, OCBs=oligoclonal bands.

© 2023 Ayroza Galvão Ribeiro Gomes AB et al. *JAMA Neurology.*

	Discovery cohort					Confirmation cohort					Pooled				
	MOG-IgA^{Isolated} (n=8)	MOG-IgG$^{±IgA/M}$ (n= 23)	MOG-IgG^{Isolated} (n=16)	MOG-IgG^{+IgA} (n=4)	*P*	MOG-IgA^{Isolated} (n=10)	MOG-IgG$^{±IgA/M}$ (n=58)	MOG-IgG^{Isolated} (n=47)	MOG-IgG^{+IgA} (n=11)	*P*	MOG-IgA^{Isolated} (n=18)	MOG-IgG$^{±IgA/M}$ (n= 81)	MOG-IgG^{Isolated} (n=63)	MOG-IgG^{+IgA} (n=15)	*P*
Female, n (%)	4 (50.0)	6 (25.0)	4 (25.0)	1 (25.0)	ns	7 (70.0)	34 (57.6)	29 (61.7)	5 (45.4)	ns	11 (61.1)	40 (49.4)	33 (52.4)	6 (40.0)	ns
Age at disease onset, y, median (range)	36.55 (22-76)	32 (6-65)	35.5 (9-65)	20.5 (6-40)	ns	26.5 (3-54)	34 (3-68)	35 (3-62)	33 (10-68)	ns	32.5 (3-76)	34 (3-68)	36 (3-65)	28 (6-68)	ns
EDSS at sampling, median (range)	2.5 (0-9.5)	2.5 (0-8.5)	2.75 (1-8.5)	2 (0-6)	ns	3 (0-6.0)	2 (0-7.5)	2 (0-7.5)	1.75 (0-6)	ns	2.75 (0-9.5)	2 (0-8.5)	2 (0-8.5)	2 (0-6)	ns
No. of attacks at last follow-up, median (range)	1.5 (1-3)	1 (1-14)	1.5 (1-14)	3 (1-9)	ns	2 (1-4)	2 (1-8)	2 (1-8)	2 (1-5)	ns	2 (1-4)	2 (1-14)	2 (1-14)	2 (1-9)	ns
Time between first attack and sampling, m, median (range)	1 (0-108)	6 (0-303)	10 (0-303)	109.5 (0-282)†	ns	43 (3-449)	64 (0-511)	44 (0-286)	115 (1-511)	ns	24 (0-449)	44 (0-511)	44 (0-303)	115 (0-511)	ns
Duration of follow-up, m, median (range)	6.5 (0-32)	18.5 (0-128)	22.5 (0-104)	9.5 (0-73)	ns	61.5 (0-108)	49 (0-227)	47 (0-227)	50 (11-116)	ns	25 (0-108)	43 (0-227)	43.5 (0-227)	42 (0-116)	ns
CSF-specific OCBs, n (%)	2/8 (25.0)	3/16 (18.8)	3/13 (23.0)	0/1 (0)	ns	3/8 (37.5)	13/32 (40.6)	11/28 (39.3)	2/4(50.0)	ns	5/16 (31.3)	16/48 (33.3)	14/41 (34.1)	2/5 (40.0)	ns
Coexisting MOG-Ig antibodies, n (%)															
MOG-IgA	NA	4 (25.0)	NA	NA	NA	NA	11 (18.6)	NA		NA	NA	15 (18.5)	NA	NA	NA
MOG-IgM	NA	3 (13.0)	NA	NA	NA	NA	0 (0)	NA		NA	NA	3 (3.7)	NA	NA	NA

© 2023 Ayroza Galvão Ribeiro Gomes AB et al. *JAMA Neurology.*

eTable 2: Demographic and clinical features of MOG-IgA and -IgG seropositive patients of the discovery, confirmation, and combined cohorts. MOG-IgA^{Isolated} refers to isolated MOG-IgA seropositive patients, MOG-IgG$^{\pm\text{IgA/M}}$ refers to MOG-IgG seropositive patients regardless of co-existence of MOG-IgA and/or -IgM. MOG-IgG^{Isolated} refers to isolated MOG-IgG seropositive patients. MOG-Ig^{Seronegative} refers to MOG-Ig seronegative patients. *P*=p-value with one-way ANOVA, EDSS=Expanded Disability Status Scale, OCBs=oligoclonal bands, CSF=cerebrospinal fluid. † $p<0.05$ in pairwise analysis with MOG-IgA group (unpaired t test).

© 2023 Ayroza Galvão Ribeiro Gomes AB et al. *JAMA Neurology.*

ID	Center	Age at disease onset,y / biological sex	Age at sampling, y	Disease manifestation	Current diagnosis	Fulfils McDonalds 2017/ICDC 2015 criteria	Time of follow up (months)	No. of attacks	EDSS at sampling	Treatment at sampling (including 90 days prior)	CSF cell count (RV <5)	CSF protein (RV 150-500)(mg/L)	CSF OCBs	Other serum antibodies	MOG-IgA (>2.375) MFI	MOG-IgG (>3.0) MFI	MOG-IgM (> 1.625) MFI	AQP-IgG (> 1:20)
1	Basel	Mid-20s/F	Mid-30s	Recurrent brainstem syndrome	RRMS	Yes/No	0	2	1.5	IFN beta-1a	N/A	N/A	N/A	N/A	3.43	1.19	1.12	<1:20
2	Basel	Mid-30s/F	Mid-30s	Recurrent short myelites	RRMS	Yes/No	32	3	2.5	None	12	425 (normal)	Type II	Rubella IgG, VZV igG	4.33	1.64	0.82	<1:20
3	Basel	Early-20s/M	Early-20s	Monophasic brainstem syndrome	Atypical demyelinating syndrome	No/No	6	N/A	0	None	4	247 (normal)	Type I	Rubella IgG, Toxoplasmosis IgG, HAV IgG, EBV IgG, VZV IgG, HSV I+II IgM, Measles IgG, Mumps IgM	3.78	1.63	1.07	<1:20
4	Basel	Late-30s/F	Late-30s	Monophasic brainstem syndrome + transient myelitis	NMOSD (seronegative)	No/Yes	20	1	1.5	PRED (10mg/day)	8	201 (normal)	Type I	HBsAg, HBc, Rubella IgG, EBV IgG, HSV I+II IgG, VZV IgG	4.18	1.43	0.97	<1:20
5	Basel	Early 60s/M	Early-60s	Brainstem syndrome + LETM + cerebral syndrome	Seronegative NMOSD and diffuse cerebral B cell lymphoma	No/Yes	7	N/A	9.5	None	9	500 (normal)	Type I	VZV IgG, HSV I+II IgG, CMV IgG, Masles IgG. Mumps IgG, Rubeolla IgG, FSME IgG. , Toxoplasmosis IgG	2.80	1.39	0.97	<1:20

© 2023 Ayroza Galvão Ribeiro Gomes AB et al. *JAMA Neurology.*

6	Basel	Late-50s/M	Early-60s	LETM	Suspected parainfectious LETM (neurocysticercosis)	No/No	30	3	2.5	AZA, PRED (35mg/day)	92	4379 (elevated)	Type III	Rubella IgG, EBV IgG, VZV IgG	4.03	1.19	0.89	<1:20
7	Basel	Mid-70s/M	Mid-70s	Symptomatic cerebral syndrome	Atypical demyelinating syndrome	No/No	3	1	4	None	3	1056 (elevated)	Type I	Measles IgG, VZV IgG, CMV IgG	5.23	1.29	1.02	<1:20
8	Basel	Early-30s/F	Early-30s	Monophasic short myelitis	RRMS	Yes/No	0	1	3.5	None	1	186 (normal)	Type I	Measles IgG, Mumps IgG, VZV IgG, Borrelia IgM, Anti-Cardiolipin IgG, Solubles IL-2	3.86	1.60	1.02	<1:20
9	Sao Paulo	Early-teens/M	Mid-teens	Monophasic bilateral optic neuritis + pachymeningitis	Atypical demyelinating syndrome	No/No	10	1	4	AZA, PRED (5mg/day)	2	250 (normal)	Type I	HSV I+II IgG	3.98	1.44	1.05	<1:20
10	Bochum	Toddler/F	Late-teens	ADEM	ADEM	No/No	43	2	1	None	3	223 (normal)	Type I	N/A	6.14	2.66 **	1.2	Negative*
11	Düsseldorf	Late-30s/F	Late-30s	Monophasic optic neuritis + LETM + short myelitis	NMOSD (seronegative)	No/Yes	15	1	1	IVMP, IVIG, PLEX, RTX	140	663 (elevated)	Type I	N/A	6.55	1.13	1.21	<1:10
12	SMSC	Mid-teens/F	Early-50s	N/A	RRMS	Yes/No	104	2	6	NTZ	NA	NA	NA	VZV IgG	2.69	1.12	1.00	NA
13	SMSC	Late-40s/F	Early-50s	N/A	RRMS	Yes/No	88	4	3	None	2.7	246	Type I	VZV IgG. Mumps IgG, Measles IgG	5.65	1.68	0.94	< 1:10
14	SMSC	Late-20s/F	Late-20s	N/A	RRMS	Yes/No	78	3	0	N/A	5.6	320	Type II	None	2.51	0.97	1.05	< 1:10
15	SMSC	Mid-20s/F	Late-20s	N/A	RRMS	Yes/No	0	3	3	IFN beta-1a	2	403	Type I	VZV IgG	2.71	1.18	1.09	NA
16	SMSC	Late-40s/M	Early-60s	N/A	PPMS	Yes/No	82	2	4.5	N/A	NA	NA	Type II	None	2.77	2.63	1.07	NA

© 2023 Ayroza Galvão Ribeiro Gomes AB et al. *JAMA Neurology.*

17	SMSC	Mid-50s/F	Late-50s	N/A	PPMS	Yes/No	45	1	4	OCR	1	330	Type I	ANA	2.50	1.03	1.06	NA
18	SMSC	Mid-20s/M	Early-30s	N/A	RRMS	Yes/No	108	3	0	FYN	28.7	730	Type II	VZV IgG	2.41	2.02	1.11	NA

eTable 3: Clinical characteristics of MOG-IgA seropositive patients.

*Data from external service. Unavailable raw values/** Borderline. ID=patient ID. ICDC=International consensus diagnostic criteria for neuromyelitis optica spectrum disorders, EDSS=Expanded Disability Status Scale, CSF=Cerebrospinal fluid, OCBs=Oligoclonal bands, IFN=interferon, PRED=prednisone, AZA=azathioprine, RTX=Rituximab, IVMP=Intravenous methylprednisolone, IVIG=Intravenous immunoglobulin, PLEX=Plasmapheresis, NTZ=Natalizumab, OCR=Ocrelizumab, FIN=Fingolimod, SMSC=Swiss Multiple Sclerosis Cohort-Study, LETM=Longitudinally extensive transverse myelitis, ADEM=Acute disseminated encephalomyelitis, RRMS=Relapsing remitting multiple sclerosis, NMOSD= Neuromyelitis optica spectrum disorders, PPMS=Primary progressive multiple sclerosis, VZV=Varicella-Zoster virus, HAV=Hepatitis A virus, EBV=Epstein-Barr virus, HSV=Herpes simplex virus, HBsAg=Hepatitis B surface antigen, HBc=Hepatitis B core antigen, CMV=Cytomegalovirus, FSME=Tick-borne encephalitis, ANA=Antinuclear antibodies.

© 2023 Ayroza Galvão Ribeiro Gomes AB et al. *JAMA Neurology.*

eReferences

1. Lennon PVA, Wingerchuk DM, Kryzer TJ, et al. A serum autoantibody marker of neuromyelitis optica: Distinction from multiple sclerosis. *Lancet*. Published online 2004. doi:10.1016/S0140-6736(04)17551-X
2. O'Connor KC, McLaughlin KA, De Jager PL, et al. Self-antigen tetramers discriminate between myelin autoantibodies to native or denatured protein. *Nat Med*. 2007;13(2):211-217. doi:10.1038/nm1488
3. Pröbstel AK, Dornmair K, Bittner R, et al. Antibodies to MOG are transient in childhood acute disseminated encephalomyelitis. *Neurology*. 2011;77(6):580-588. doi:10.1212/WNL.0b013e318228c0b1
4. Marignier R, Hacohen Y, Cobo-Calvo A, et al. Myelin-oligodendrocyte glycoprotein antibody-associated disease. *Lancet Neurol*. 2021;20(9):762-772. doi:10.1016/S1474-4422(21)00218-0
5. Pröbstel AK, Rudolf G, Dornmair K, et al. Anti-MOG antibodies are present in a subgroup of patients with a neuromyelitis optica phenotype. *J Neuroinflammation*. 2015;12(46). doi:10.1186/s12974-015-0256-1
6. Wingerchuk DM, Banwell B, Bennett JL, et al. International consensus diagnostic criteria for neuromyelitis optica spectrum disorders. *Neurology*. Published online 2015. doi:10.1212/WNL.0000000000001729
7. Sabatino JJ, Pröbstel AK, Zamvil SS. B cells in autoimmune and neurodegenerative central nervous system diseases. *Nat Rev Neurosci*. 2019;20(12):728-745. doi:10.1038/s41583-019-0233-2
8. Banwell B, Bennett JL, Marignier R, et al. Diagnosis of myelin oligodendrocyte glycoprotein antibody-associated disease: International MOGAD Panel proposed criteria. *Lancet Neurol*. 2023;22(3):268-282. doi:10.1016/S1474-4422(22)00431-8
9. Breedveld A, van Egmond M. IgA and FcαRI: Pathological Roles and Therapeutic Opportunities. *Front Immunol*. 2019;10. doi:10.3389/fimmu.2019.00553
10. Pröbstel AK, Zhou X, Baumann R, et al. Gut microbiota–specific iga+ B cells traffic to the CNS in active multiple sclerosis. *Sci Immunol*. 2020;5(53). doi:10.1126/SCIIMMUNOL.ABC7191
11. Thompson AJ, Banwell BL, Barkhof F, et al. Diagnosis of multiple sclerosis: 2017 revisions of the McDonald criteria. *Lancet Neurol*. 2018;17(2):162-173. doi:10.1016/S1474-4422(17)30470-2
12. Pedreño M, Sepúlveda M, Armangué T, et al. Frequency and relevance of IgM, and IgA antibodies against MOG in MOG-IgG-associated disease. *Mult Scler Relat Disord*. 2019;28:230-234. doi:10.1016/j.msard.2019.01.007

© 2023 Ayroza Galvão Ribeiro Gomes AB et al. *JAMA Neurology.*

13. Schumacher H, Wenke NK, Kreye J, et al. IgA autoantibodies against native myelin basic protein in a patient with MS. *Neurology(R) neuroimmunology & neuroinflammation*. 2019;6(4):e569. doi:10.1212/NXI.0000000000000569
14. Prüss H, Höltje M, Maier N, et al. IgA NMDA receptor antibodies are markers of synaptic immunity in slow cognitive impairment. *Neurology*. 2012;78(22):1743-1753. doi:10.1212/WNL.0b013e318258300d
15. Wilhelm I, Nyúl-Tóth Á, Suciu M, Hermenean A, Krizbai IA. Heterogeneity of the blood-brain barrier. *Tissue Barriers*. 2016;4(1). doi:10.1080/21688370.2016.1143544
16. Disanto G, Benkert P, Lorscheider J, et al. The Swiss Multiple Sclerosis Cohort-Study (SMSC): A Prospective Swiss Wide Investigation of Key Phases in Disease Evolution and New Treatment Options. *PLoS One*. 2016;11(3):e0152347. doi:10.1371/journal.pone.0152347
17. Kunchok A, Chen JJ, McKeon A, Mills JR, Flanagan EP, Pittock SJ. Coexistence of Myelin Oligodendrocyte Glycoprotein and Aquaporin-4 Antibodies in Adult and Pediatric Patients. *JAMA Neurol*. 2020;77(2):257. doi:10.1001/jamaneurol.2019.3656
18. Filippi M, Preziosa P, Banwell BL, et al. Assessment of lesions on magnetic resonance imaging in multiple sclerosis: practical guidelines. *Brain*. 2019;142(7):1858-1875. doi:10.1093/brain/awz144
19. Cagol A, Fuertes NC, Stoessel M, et al. Optical coherence tomography reflects clinically relevant gray matter damage in patients with multiple sclerosis. *J Neurol*. Published online January 10, 2023. doi:10.1007/s00415-022-11535-8
20. Schippling S, Balk L, Costello F, et al. Quality control for retinal OCT in multiple sclerosis: validation of the OSCAR-IB criteria. *Multiple Sclerosis Journal*. 2015;21(2):163-170. doi:10.1177/1352458514538110
21. Linnington C, Webb M, Woodhams PL. A novel myelin-associated glycoprotein defined by a mouse monoclonal antibody. *J Neuroimmunol*. 1984;6(6):387-396. doi:10.1016/0165-5728(84)90064-X

© 2023 Ayroza Galvão Ribeiro Gomes AB et al. *JAMA Neurology.*
