# Supplementary material for: Immunoglobulin A Antibodies Against Myelin Oligodendrocyte Glycoprotein in a Subgroup of Patients With Central Nervous System Demyelination
Source: JAMA Neurol. 2023 Aug 7;80(9):989–95. doi: 10.1001/jamaneurol.2023.2523 (PMC10407763; doi:10.1001/jamaneurol.2023.2523)
Supplement: Supplement 2. — Data sharing statement [file jamaneurol-e232523-s002.pdf]

Data Sharing Statement

Ayroza Galvão Ribeiro Gomes. Immunoglobulin A Antibodies Against Myelin Oligodendrocyte Glycoprotein in a Subgroup of Patients With Central Nervous System Demyelination. *JAMA Neurol*. Published August 07, 2023. doi:10.1001/jamaneurol.2023.2523

Data
Data available: No

Additional Information
Explanation for why data not available: Data will be shared upon request by the corresponding author within the limitations provided by the ethical committee in regard to individual patient data.
